# Cell Bioprinting: The 3D-Bioplotter™ Case

**DOI:** 10.3390/ma12234005

**Published:** 2019-12-02

**Authors:** David Angelats Lobo, Paola Ginestra

**Affiliations:** 1 Department of Mechanical and Industrial Engineering, University of Brescia, V. Branze 38, 25123 Brescia, Italy; paola.ginestra@unibs.it; 2 New Therapeutic Targets Laboratory (TargetsLab)-Oncology Unit, Department of Medical Sciences, Faculty of Medicine, University of Girona, Emili Grahit 77, 17003 Girona, Spain

**Keywords:** 3D printing, biopolymers, bioprinting

## Abstract

The classic cell culture involves the use of support in two dimensions, such as a well plate or a Petri dish, that allows the culture of different types of cells. However, this technique does not mimic the natural microenvironment where the cells are exposed to. To solve that, three-dimensional bioprinting techniques were implemented, which involves the use of biopolymers and/or synthetic materials and cells. Because of a lack of information between data sources, the objective of this review paper is, to sum up, all the available information on the topic of bioprinting and to help researchers with the problematics with 3D bioprinters, such as the 3D-Bioplotter™. The 3D-Bioplotter™ has been used in the pre-clinical field since 2000 and could allow the printing of more than one material at the same time, and therefore to increase the complexity of the 3D structure manufactured. It is also very precise with maximum flexibility and a user-friendly and stable software that allows the optimization of the bioprinting process on the technological point of view. Different applications have resulted from the research on this field, mainly focused on regenerative medicine, but the lack of information and/or the possible misunderstandings between papers makes the reproducibility of the tests difficult. Nowadays, the 3D Bioprinting is evolving into another technology called 4D Bioprinting, which promises to be the next step in the bioprinting field and might promote great applications in the future.

## 1. Introduction

Three-dimensional (3D) printing, also called Rapid Prototyping (RP), was originally developed by Charles Hull in 1986 as a technique called stereolithography (SLA) [1,2]. For being the first 3D technology ever conceived, its precision and resolution were and are still high [3].

**Figure 1:**
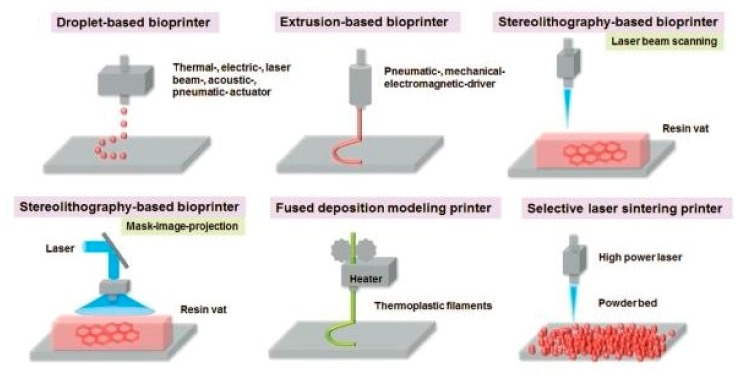
Examples of the available techniques in the 3D printing field [12].

The first technology was stereolithography, which consists of the solidification of a photosensitive material by an ultraviolet light source [4]. Later, other 3D printing techniques were conceived such as fused deposition modelling (FDM) [5], inkjet printing, direct laser patterning, cell-sheet technology, cell-laden technology, extrusion-based printing [6], valve-based technology, acoustic printing [7], selective laser melting [8], selective laser sintering [9], and laminated object manufacturing [10]. Some of these technologies can be seen in Figure 1. All of them can also be classified into four different categories, like extrusion printing, material sintering, material binding, and lamination [11].

Those technologies were first applied in the 3D printing field, but, 17 years ago, a new field was introduced called 3D Bioprinting, and the first application was the development of vascular tissue networks to maintain the cells within culture [13]. In addition, another application was the production of synthetic biocompatible supports for cells, also called scaffolds, to mimic the natural cellular microenvironment [14]. Several conditions must be accomplished before bioprinting, such as the acquisition of a 3D image, a computer-aided design (CAD) software [15], and the ability to control the deposition of the materials used [16].

Different approaches can be used to bioprint, either with or without cells at the initial step [12]. In particular, 80% of printers are optimized for an extrusion-based printing [17]. The material extrusion, especially of thermoplastic materials, is the most common and inexpensive technique because it can use a wide range of materials like polylactic acid (PLA), polycaprolactone (PCL), polyvinyl alcohol (PVA), and biodegradable calcium phosphate glass, which are then combined with cells such as human monocytes, for example to study the inflammation process [18]. On the other hand, the bioprinting technique can use cells directly so the design of a proper structure for the accommodation of cells in the synthesized scaffolds is more complicated but offers some advantages such as the possibility to optimize the cell deposition and distribution, and the printing speed [11]. Thus, the main difference between a typical material extrusion and a bioprinting technique is that the first one does not use cells directly, so it requires a post-seeding process that might not be required for bioprinting techniques.

As previously mentioned, the bioprinting process can be performed using two different approaches, called pre-seeding and post-seeding [19]. The pre-seeding bioprinting is a type of 3D bioprinting that involves the printing of both materials and cells at the same time. Although it requires more time to properly optimize the geometry of the scaffold manufactured, it also provides high applicability and efficiency. On the other hand, the post-seeding process, which could be used after an extrusion-based printing, consists of first printing the material and then co-culturing it with the proper cells. In this review paper, those techniques are related to the step in which the extrusion material and the cells are combined, as it could be at the same time for direct bioprinting, or after the printing of the material (i.e., mold or sacrificial structure) for indirect bioprinting. Compared to the direct bioprinting, the indirect one has lower efficiency. To sum up, direct bioprinting is more time-consuming than indirect bioprinting, but it also has higher efficiency on cell deposition and might also be a way to increase cell viability within the scaffold designed by not exposing cells under more stress.

In that context, several combinations of materials and cells, also called bio-inks, can be used to perform direct bioprinting by combining materials such as microcarriers, decellularized extracellular matrixes (dECM), and hydrogels with cells from tissue spheroids, cell pellets, and tissue strands [20].

Specifically, hydrogels have some interesting properties because they are in a solid/aqueous state. They are easily controllable by changing temperature and humidity [21], biodegradable, biocompatible, with tailorable mechanical strength, and readily available [17]. Their limits are related to the dissolution kinetics in body fluids and the difficult sterilization process. Of course, other materials can be used to avoid these limitations such as metals and metal alloys, ceramics and carbon compounds, and composites [11].

The most important bioprinting limitations are connected with the need of a vascular network to maintain cell viability within the bioprinted tissue or organ [7], the presence of bottlenecks between biology and engineering to bioprint complex compositions [22,23], the complexity of native tissues [24], the viscosity of the material [25,26,27], and, finally, the bio-inks available on the market. An ideal bio-ink must be strongly biocompatible, with appropriate rheological parameters [15,28], architectural integrity, and assure an equilibrium between cell viability and functionality after bioprinting [29].

In this review, we will focus on applications of 3D bio-printers available on the market, mainly the 3D-Bioplotter™ systems, for both direct and indirect bioprinting. We will be focused on 3D-Bioplotter™ systems because of their precision, flexibility, and user-friendly employment. These printers also offer the possibility of a process optimization in relation to the effects of the parameters and their interdependence with a stable platform that leads to a higher replicability of the results compared to other bioprinters available on the market. Moreover, we will refer to the state of the art on bioprinting, what has been done, and what will be needed for future studies.

## 2. Materials for Bioprinting

### 2.1. Polymers

#### 2.1.1. Natural Polymers

Natural polymers, also called biopolymers, have different properties and advantages, related to their chemical-physical compositions that can be adjusted to the target tissue and cell types [30,31,32]. If the scaffolds are properly planned, cells can have enough space for cell proliferation and migration [33]. Rheological parameters also need to be considered because they have high relevancy for the biofabrication process. Some of those parameters are the viscosity, shear-thinning, yield stress, and porosity, among others [34]. The use of biopolymers allows a better mimicry of the natural microenvironment of cells but have reproducibility problems of experiments because of their batch-to-batch variability.

Nowadays, bioprinting uses many natural and semi-synthetic polymers, such as collagen and fibrinogen [35,36], gelatin methacrylamide (GelMA) [34,37], alginate [38,39], Matrigel™ and Cultrex^®^ [40], and basement membranes (ECM containing proteins like fibronectin, laminin, and collagen type IV) [41,42]. Other strategies can include the acquisition of ECM by inducing it to a chondrocyte culture and then separating it from the cells by a devitalized technique [43].

#### 2.1.2. Synthetic Polymers

Internal variations on natural polymer synthesis make the comparison between experiments difficult. Synthetic polymers solve that problem because they have an exact structure and composition between samples.

Some of the materials that are used might be polycaprolactone (PCL), polyethylene glycol and derivatives (PEG), polylactic acid (PLA), poly(lactic-co-glycolic acid) (PLGA) [44,45], and peptide scaffolds of BD™ Pura Matrix™ [41,46].

### 2.2. Cross-Linking Methods

**Figure 2:**
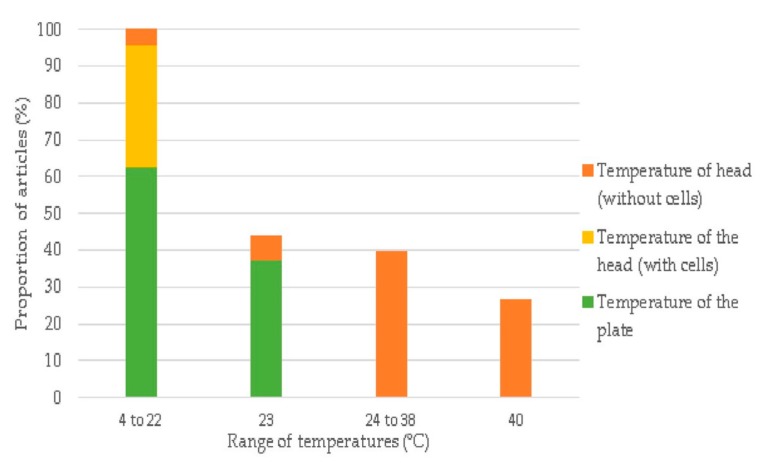
Description of the cross-linking techniques and their materials used for the 3D-Bioplotter™ bioprinter. The information is represented as percentages (%) and the different materials used are represented by colors.

Different cross-linking methods can be employed to retain certain geometries of the materials used for bioprinting, such as chemical, light, physical, and hybrid techniques. As seen in Figure 2, each material used is classified according to the type of cross-linking method used. As for the chemical cross-linking, the majority of the articles use alginate as the main material (80%) followed by alginate: gelatin (15%) and PEG-polymers (5%). In relation to the light techniques, the majority of the articles report the use of methacrylated-gelatin (55.6%), followed by methacrylated hyaluronic acid (33.3%), and hydroxyapatite (11.1%) as main materials of the hydrogels. Concerning the hybrid techniques, for the combination of more than one technique, the majority of the authors use methacrylated-gelatin (75%) or alginate (25%). Respecting the physical techniques, by the use of temperature, for example, the majority of the articles report as main material lignin combined with HPU (50%) or decellularized ECM (50%). Finally, a small part of the articles does not specify the cross-linking method used, and the main materials used for their hydrogels are alginate (40%), alginate: gelatin (40%) or methacrylated-gelatin (20%). Some examples of each category could be, for the chemical methods, the use of calcium ion solution, for light techniques, the exposure to a UV light source, physical cross-linking by temperature and hybrid techniques, by using more than one technique at the same time.

### 2.3. Cellular Typologies

In this section, the most important cell types that are being used with different types of 3D bioprinters, and with the 3D-Bioplotter™ will be described. All of the available information will be distributed in subsections according to the type of cells used.

#### 2.3.1. Vascular Tissues

Vascularization is very important for the bioprinting, especially for large tissue constructs, because cells need a constant supply of nutrients and oxygen [12]. According to analyzed literature, one of the strategies is the use of the HUVEC cell line to develop vascular networks for cell viability maintenance, as seen in Table 1.

It seems that the presence of a PEG-derived polymer within the hydrogel is needed for the establishment of a proper vascular network for cell viability preservation. Because there is a lack of information on the cell line used with a modified thermal inkjet printer, we cannot confirm that the use of a sacrificial material like carbohydrate glass filament networks could be a better alternative than using PEG-derived polymers for microvascular network synthesis.

#### 2.3.2. Cartilage and Bone-Like Structures

Many cartilage and bone applications can be reflected in Table 2, by 3D-Bioplotter™ and other 3D bioprinting machines. For cartilage tissue engineering, the majority of the cells used are related to primary chondrocytes followed by one example of human chondrocytes [51]. For bone tissue engineering, there are only two examples in the table, by using bone marrow stromal cells (BMSCs) combined with different hydrogels and endothelial stromal cells derived from the stromal vascular fraction of adipose tissue (SVFCs) used for the prevascularization process of bone constructs. The majority of articles are focused on regenerative medicine applications such as improvement on bioprinted cartilage [52], orthopedics [53,54,55], bone tissue bioprinting [56], prevascularization on bone tissue constructs [57] and cartilage tissue engineering [58,59]. One of the indirect contributions to the regenerative medicine field would be the study of a reversible cross-linking strategy [60]. In Table 2, there is only one example of human chondrocytes with a PEGDA hydrogel [51], which could be an isolated case because the articles that use primary chondrocytes are combined with alginate-based and/or gelatin methacryloyl hydrogels. There is also a lack of information on three articles about the cell lines used, which could help to determine if there is a relationship between the cell lines used and the compounds of their hydrogels.

#### 2.3.3. Cardiac Tissues

The principal applications for cardiac tissue engineering are reported in Table 3, mainly focused on 3D-Bioplotter™ and with only one example of another brand of 3D bioprinter. The applications are focused on the generation of tissue spheroids [61], regenerative medicine for the generation of cardiac patches [62], cardiac implants, and nano-reinforced cardiac patches’ [63].

For 3D-Bioplotter™, human cardiac progenitor cells (hCPCs) and human coronary artery endothelial tissues became used for regenerative medicine applications, combined with alginate-based hydrogel or gelatin methacrylate hydrogel, then supplemented with support materials such as cardiac extracellular matrix, PEI, calcium chloride, methacrylated collagen, and carboxyl functionalized carbon nanotubes (CNTs).

#### 2.3.4. Liver Tissues

For liver tissue applications, exposed in Table 4, there are only two examples of each type of 3D bioprinter. In the case of Organovo 3D-bioprinter, there is no information about the cell lines and materials used [64], which make the comparison of the two types of bioprinters difficult. The only described example is the use of 3D-Bioplotter™ for the bioprinting of liver tissue using a decellularized extracellular matrix of the liver and a sacrificial material called Pluronic F-127, combined with immortalized mouse small cholangiocytes and a cancer cell line called HUH7 [65]. 

#### 2.3.5. Stem Cells

In the subject of stem cell applications, the majority of the papers use an alginate-based hydrogel with only three examples of methacrylated gelatin hydrogels combined [66,67,68,69,70], as reflected in Table 5.

There is also a clear relationship between cell lines and hydrogel compositions, in some cases. This can be observed with iPS and neural stem cells that use an alginate hydrogel supplemented with carboxymethyl-chitosan and agarose, with human mesenchymal stem cells for containing methacrylated gelatin as one component of their respective hydrogels [69,70]. The majority of applications are related to regenerative medicine such as the production of neural mini-tissues [60] but also related to model development for drug testing and the study of diseases such as breast cancer [67] and preeclampsia [69]. Only two examples are related to the development of techniques such as dielectric impedance spectroscopy technique [68] and mesoscopic fluorescence tomography [70].

#### 2.3.6. Cancer Cells

Principal cancer cell applications are represented in Table 6. Alginate is the main component of the hydrogels, followed by methacrylated gelatin and a complex hydrogel formulation [67].

The applications are related to regenerative and other medical studies but only is associated with cancer study, which in that case is drug testing using an HER-2 positive breast cancer cell line called 21PT, combined with a complex hydrogel based on methacrylated gelatin supplemented with other compounds [67]. One interesting study would be the one associated with the biofabrication of constructs with high cell viability because the authors performed a photo-crosslinking technique with a UV light source that apparently does not affect the cell viability of the scaffold [74].

#### 2.3.7. Adipose Tissues

In the matter of adipose tissue applications, only two examples are found in Table 7 that use methacrylated gelatin-based hydrogels. Only one example is related to regenerative medicine on adipose tissue engineering, with supplementation of PEG-4A in the hydrogel [50]. The other application is related to the metabolic study for the differences between white and brown adipose tissues [57].

#### 2.3.8. Muscle Cells

For muscle cells, there is only one application that uses L8 myoblasts and Schwann cells, combined with an alginate-based hydrogel for a study of cell damages of bioprinting processes [78], seen in Table 8.

#### 2.3.9. Schwann Cells

Concerning Schwann cell applications, the main component of the hydrogels employed is alginate, supplemented with different support materials depending on the cell line and resulted applications. Some applications the development of better peptide-modified alginate scaffolds [79], the repair of peripheral nerve injury [80], production of scaffolds with high integrity and cell viability [81], and the explanation of cell damage and proliferative ability on bioprinting processes [78]. Further information can be found in Table 9.

#### 2.3.10. Skin Tissues

Relative to skin tissue applications (Table 10) almost all hydrogels use methacrylated gelatin followed by PEG formulations and a novel hydrogel formulation. One interesting article is the proposed alternative hydrogel formulation based on lignin, which is suggested as a new concept for skin tissue bioprinting. The majority of the papers correspond to regenerative medicine, except for one on the use of mesoscopic fluorescence tomography, previously mentioned [70]. Except for the novel formulation [82], it seems that the common cross-linking method used is the chemical one, exempting the use of a photoinitiator and tyrosinase on bioprinting of living skin tissue constructs [83]. 

### 2.4. General Summary

**Figure 3:**
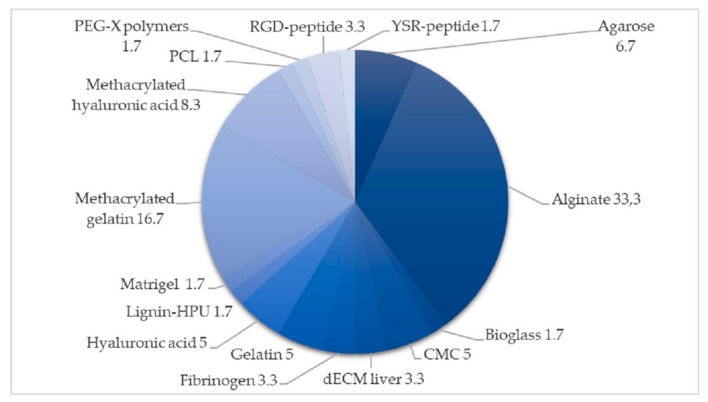
Main materials used for scaffolds bioprinting. The information is represented as percentages (%).

**Figure 4:**
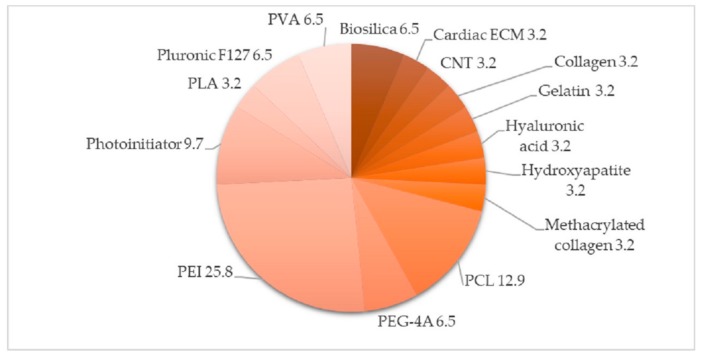
Support materials used for scaffolds. The information is represented as percentages (%).

The main and support materials used for bioprinting can be seen in Figure 3 and Figure 4, obtained from a revision of the literature of 40 articles on bioprinting tests with the 3D-Bioplotter™.

As seen in Figure 3, the most common material used for scaffold manufacturing is alginate. Alginate is a good candidate because it is cheap, easy to print, to handle and extrude while protecting encapsulated cells within it [84]. It has limits such as the absence of cell-adhesion properties [85], but they can be avoided by adding gelatin [86], hyaluronic acid [75], or methacrylated collagen [63] as support materials.

As for the scaffold geometry, there is not a clear default geometry because it depends on the type of bio-inks used and the authors and the final applications in each case. As previously said, alginate is an interesting material to be used for bioprinting, not only because it is cheap but also because of its high biocompatibility and the ability to absorb water, and therefore the ability to control cell viability within the hydrogel [87,88].

One of the main issues in 3D bioprinting is to maintain the cell viability because many factors such as shear stress during printing and cell encapsulation could reduce the cell growth from 40% to 2% and that cell damage may also be caused by the different cross-linking processes performed after bioprinting [89]. Materials by themselves, like alginate, have some limitations that might influence the cell viability during bioprinting, so the combination with support materials could be helpful to reduce these problems [90]. For example, the combination of alginate and biosilica resulted in being more promising not only for the bone tissue culture formation than alginate or biosilica alone, but also for the cell viability due to the improved extrusion process [72]. Other authors proposed the use of cylindrical cell aggregates, composed of mouse bone marrow cells (BMSC), Schwann cells (SC) and agarose, to not only make it easy to handle the bio-ink but also not affecting the generation of the proper post-printing structure because of a reduction on the cell damage [91]. When the main application is regenerative medicine and transplants, an autograft of adult stem cells, especially adipose-derived stem cells, can be used safely to avoid the rejection process during transplantation [92].

Other improvements can be made for mechanical properties such as mechanical strength, elasticity, and stiffness. Some of the strategies can be, for example, the cross-linking methods by exposure to ultraviolet light, heat, and/or an ionic solution.

## 3. Manufacturing Parameters

### 3.1. Temperature of the Head and Plate

This relationship is the most uncertain, firstly because the process temperatures are mostly related to the bioprinted materials and due to the lack of information on the temperature of the head and plate of the 3D-Bioplotter™, among the different papers consulted.

Most of the articles only contain one of those two parameters (57.5%) and only a small portion of the research (5%) includes all the available information to understand the possible correlation between them. The majority of the papers report a temperature of the head and the plate around 22 °C while printing alginate or methacrylated gelatin-based hydrogels in the presence of cells. A high portion of papers gives no indications on those parameters, which have a strong relationship with the cell survival rate in the synthesized constructs. While the temperature of the head is more related to the cells’ viability and the material properties, the temperature of the plate could be a crucial parameter because the plate is involved in processes like physical cross-linking and the maintenance of cell viability post-printing. The temperature information could be beneficial especially when newly developed materials are used.

### 3.2. Pressure

Pressure is an important parameter to be considered, not only because every polymer has its specific properties such as viscosity among others, but also because, when the printing is performed with cells, they need to be maintained all of the time in the optimal conditions because a stress situation provoked, for example, by higher pressures might be capable of altering the viability of the cells, and reducing it, which can be a problem for the experiments that are being performed. In Table 11, we can see some examples of different cell lines from bone and cartilage tissues, stem cells, cancer cells, adipose tissues, Schwann cells related to the nervous system and fibroblasts, in this order.

## 4. Applications of Bioprinting

**Figure 5:**
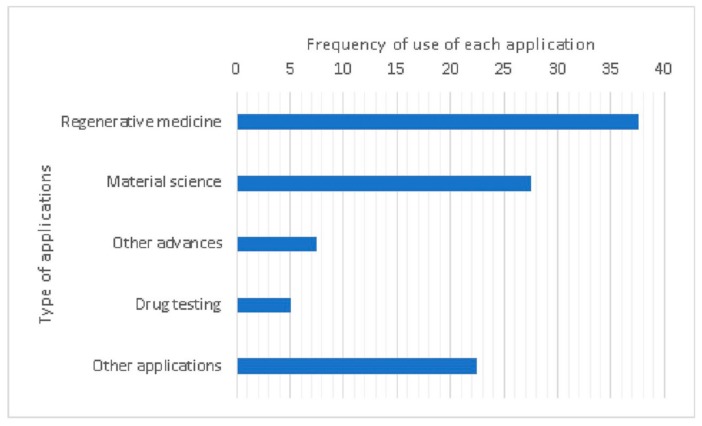
Classification of applications for the 3D-Bioplotter™ printer. All of the available information is classified into five categories, and the results are expressed as percentages (%).

The applications of bioprinting can be classified by their field, such as regenerative medicine, material science, drug testing, and other (i.e., cellular characterization). As illustrated in Figure 5, the main application in 3D bioprinting is regenerative medicine (37.5%). Some examples could be those related to the production of implants for cardiac failure, audition-loss [62,69], cartilage tissue engineering [58,93], and human neural tissue construction [7]. According to the analyzed literature, drug tests are mainly related to the design of cellular models for clinical research. Apropos of material science and other advanced applications, some examples are those related to cell-compatible hydrogel synthesis [27], improvements on cell viability maintenance during bioprinting processes [68], and the development of new materials such as a combination between lignin and polyurethane [82]. Other examples related to the medical field, classified as other applications, are cellular characterization [94], chemical material characterization [95], development of new techniques [70,76], and gene characterization [77].

## 5. Discussion

As previously mentioned, different materials and systems can be used for 3D bioprinting, and especially for regenerative medicine. Focusing on 3D-Bioplotter™ systems, the main material used for scaffold manufacturing is alginate, but combined with other polymers in order to improve its mechanic and biologic properties. One of the possible improvements is the use of polyethyleneimine (PEI) as a chemical cross-linking, in order to improve the mechanical stability of the 3D constructs [80,81].

Furthermore, 37.5% of the analyzed articles do not have clear temperature information and that is very crucial for the maintenance of cell viability because variations of those parameters can increase cell viability and, therefore, affect the validation of experiments in the 3D bioprinting field.

Furthermore, a new field derived from 3D bioprinting was introduced recently, called 4D bioprinting. The main difference between 3D and 4D bioprinting is that this latest technology uses smart materials that can re-shape in the response to external stimuli such as light, temperature, and humidity [96]. This new technology uses the same 3D printers but with different materials, so it is an improvement in the material science side. All the smart materials must fulfill the same properties as the biomaterials used in 3D bioprinting, such as biocompatibility, non-inflammatory response, dynamic and supporting physiological functions [97], non-toxicity, and with appropriate rheological properties if needed [98].

Thus, even though 3D bioprinting was established 17 years ago, there are still some limitations on the manufacturing processes as well as on the availability of bio-inks on the market, to mimic more exactly the natural cell microenvironment. Further studies might be developed to improve the fabrication of tissue-engineered scaffolds [83]. In the future, it will be necessary for the development of high-resolution multi-material bioprinters and accurate stimulation methods to be used not only in a regenerative medicine field but also in research in general, in order to find new biomarkers on more diseases or disorders, and help treat them more effectively.

## Figures and Tables

**Table 1:** Principal vascular tissue applications.

3D Printer Used	Cell Line (s) Used	Materials Used	Application	Reference
Modified thermal inkjet printer from HP^®^ and Canon^®^	Not specified	Sacrificial material (carbohydrate glass filament networks)	Microvascular networks	[47,48]
3D-Bioplotter™	HUVEC ^1^	Gelatin ink completed with PEG-SVA	Cell-compatible hydrogels	[49]
3D-Bioplotter™	HUVEC and HWA ^2^	Methacrylated gelatin, methacrylated hyaluronic acid, and PEG-4A ^3^	Robust cryogel for adipose tissue engineering	[50]

^1^ human umbilical vein endothelial cells. ^2^ human adipose progenitor cell line. ^3^ polyethylene glycol-valeric acid.

**Table 2:** Principal cartilage and bone applications.

3D Printer Used	Cell Line (s) Used	Materials Used	Applications	Reference
Modified HP^®^ Deskjet 500 printer	Human chondrocytes	PEGDA ^1^ hydrogel	Ambiguous	[51]
Multihead deposition system (MHDS) printer from AM technology	Not specified	Alginate-based ink completed with PCL ^2^	Strength improvement on bioprinted cartilage	[52]
3D printer	Not specified	PCL/hydroxyapatite hydrogel	Orthopaedic applications	[32,34,53,54]
Biological laser (BioLP) printer designed in the laboratory	Not specified	Alginate/hydroxyapatite hydrogel	Orthopedic applications	[55]
3D-Bioplotter™	BMSCs ^3^	Non-medical alginate hydrogel and calcium chloride/Lutrol F127/Matrigel/Agarose and methylcellulose	Patterned constructs for bone tissue bioprinting	[56]
3D-Bioplotter™	SVFC ^4^	PCL/hydroxyapatite hydrogel	Prevascularization in 3D bioprinted bone constructs	[57]
3D-Bioplotter™	Primary chondrocytes, other cells	Alginate hydrogel, PCL and calcium chloride	Cartilage tissue engineering	[58]
3D-Bioplotter™	Primary chondrocytes	Alginate/hydroxyapatite hydrogel	Cartilage tissue engineering	[59]
3D-Bioplotter™	Primary chondrocytes, Mesenchymal stem cells, Cartilage derived progenitor cells	Gelatin methacryloyl hydrogel, with a photoinitiator	Reversible cross-linking strategy on cartilage tissue engineering	[60]

^1^ poly(ethylene glycol) diacrylate. ^2^ polycaprolactone. ^3^ bone marrow stromal cells. ^4^ endothelial stromal cells derived from the stromal vascular fraction of adipose tissue.

**Table 3:** Principal cardiac tissue applications.

3D Printer Used	Cell Line (s) Used	Materials Used	Applications	Reference
Printer designed by nScrypt Inc.	Cardiac cells and HUVEC ^1^	Not specified	Tissue spheroids	[61]
3D-Bioplotter™	hCPCs ^2^	Gelatin methacrylate hydrogel and cardiac ECM ^3^	Cardiac patches	[62]
3D-Bioplotter™	Human coronary artery endothelial tissues	Alginate hydrogel and calcium chloride/PEI ^4^	Cardiac implants	[63]
3D-Bioplotter™	Human coronary artery endothelial cells	Alginate hydrogel and methacrylated collagen and CNTs ^5^	Nano-reinforced cardiac patches	[63]

^1^ human umbilical vein endothelial cells. ^2^ human cardiac progenitor cells. ^3^ extracellular matrix. ^4^ polyethyleneimine. ^5^ carboxyl functionalized carbon nanotubes.

**Table 4:** Principal liver tissue applications.

3D Printer Used	Cell Line (s) Used	Materials Used	Applications	Reference
Organovo 3D-bioprinter	Not specified	Not specified (with problems)	Microliver tissues for in vitro drug testing	[64]
3D-Bioplotter™	Immortalized mouse small cholangiocytes and HUH7 ^1^	dECM ^2^ of the liver and sacrificial material (Pluronic F-127)	3D-Bioprinting for liver tissues	[65]

^1^ human hepatocellular carcinoma cell line. ^2^ decellularized extracellular matrix.

**Table 5:** Principal stem cell applications.

3D Printer Used	Cell Line (s) Used	Materials Used	Applications	Reference
3D-Bioplotter™	iPSCs ^1^ and/or hNSCs ^2^	Alginate-CMC ^3^ hydrogel	Tissue bioprinting	[60]
3D-Bioplotter™	iPSCs	Alginate-CMC-agarose hydrogel and calcium chloride	In situ cell proliferation and successive multilineage differentiation	[66]
3D-Bioplotter™	ASMCs ^4^	Complex hydrogel (methacrylated hyaluronic acid, methacrylated gelatin, hyaluronic acid and gelatin	Breast cancer model for drug resistance study	[67]
3D-Bioplotter™	Human mesenchymal stem cells	Methacrylated gelatin hydrogel	Placenta model for preeclampsia	[69]
3D-Bioplotter™	Frontal cortical human neural stem cells	Alginate-CMC-agarose hydrogel and calcium chloride	Human neural tissues’ applications	[60]
3D-Bioplotter™	Frontal cortical human neural stem cells	Alginate-CMC-agarose hydrogel and calcium chloride	Production of neural mini-tissues	[60]
3D-Bioplotter™	Human mesenchymal stem cells and L929 fibroblasts	Gelatin methacrylate hydrogel/alginate hydrogel and calcium chloride	Mesoscopic fluorescence tomography for bone tissue engineering	[70]
3D-Bioplotter™	hASCs ^5^	Alginate hydrogel and calcium chloride	Monitoring of 3D constructs via dielectric impedance spectroscopy technique	[68]
3D-Bioplotter™	Human adipose-derived mesenchymal stem cells	Sodium alginate-gelatin hydrogel	Osteogenesis’ applications on in vivo studies	[71]

^1^ induced-pluripotent stem cells. ^2^ human neural stem cells. ^3^ carboxymethyl-chitosan. ^4^ adipose-derived mesenchymal stem/stromal cells. ^5^ human adipose-derived stem cells.

**Table 6:** Principal cancer cell applications.

3D Printer Used	Cell line (s) Used	Materials Used	Applications	Reference
3D-Bioplotter™	21PT cell line ^1^	Complex hydrogel (methacrylated hyaluronic acid, methacrylated gelatin, hyaluronic acid and gelatin	Breast cancer model for drug resistance study	[67]
3D-Bioplotter™	SaOS-2 cell line ^2^	Biocalcite hydrogel (alginate and biosilica)	Synthesis of calcium phosphate-bone	[72]
3D-Bioplotter™	HUH7 ^3^ and immortalized mouse small cholangiocytes	dECM ^4^ of the liver and sacrificial material (Pluronic F-127)	3D-Bioprinting for liver tissues	[65]
3D-Bioplotter™	SaOS-2 cell line	Alginate-gelatin-bioglass hydrogel, polyP/calcium chloride, and silica/biosilica	Growth and biomineralization of SaOS-2 cells on bioglass	[72]
3D-Bioplotter™	SaOS-2 cell line	Alginate-gelatin-agarose hydrogel and calcium chloride	Bioprinting of bioartificial tissue	[73]
3D-Bioplotter™	MG63 cell line ^5^ and hASCs ^6^	Alginate hydrogel and calcium chloride	Monitoring of 3D constructs via dielectric impedance spectroscopy technique	[68]
3D-Bioplotter™	HepG2 ^7^	Methacrylated gelatin B-type photocurable with UV-light	Constructs with high cell viability	[74]
3D-Bioplotter™	ATDC5 ^8^	Alginate hydrogel and PCL ^9^	Cartilage tissue engineering	[58]
3D-Bioplotter™	ATDC5	Alginate-hyaluronic acid hydrogel and calcium chloride or PVA ^10^ or PEI ^11^	Tissue reparation	[75]
3D-Bioplotter™	ATDC5	Alginate hydrogel and PCL and calcium chloride	Cartilage tissues’ applications	[76]
3D-Bioplotter™	JEG3 cell line ^12^ and trophoblast cells	Methacrylated gelatin hydrogel and EGF ^13^	Testing on ZEB2, a master regulator of EMT ^14^	[77]

^1^ HER2 -positive breast tumour cell line. ^2^ sarcoma osteogenic cell line. ^3^ hepatocellular carcinoma cell line. ^4^ decellularized extracellular matrix. ^5^ osteosarcoma cell line. ^6^ human adipose-derived stem cells. ^7^ hepatocarcinoma cell line. ^8^ mouse teratocarcinoma cell line. ^9^ polycaprolactone. ^10^ poly(vinyl alcohol). ^11^ polyethyleneimine. ^12^ choriocarcinoma cell line. ^13^ epidermal growth factor. ^14^ epithelial-mesenchymal transition.

**Table 7:** Principal adipose tissue applications.

3D Printer Used	Cell Line (s) Used	Materials Used	Applications	Reference
3D-Bioplotter™	WAP ^1^ and BAP ^2^	Methacrylated hyaluronic acid-methacrylated gelatin and hyaluronic acid and gelatin	Checking behaviour and metabolic function on human brown adipocyte	[57]
3D-Bioplotter™	HWA ^3^ and HUVEC ^4^	Methacrylated gelatin, methacrylated hyaluronic acid, and PEG-4A ^5^	Robust cryogel for adipose tissue engineering	[50]

^1^ human white adipose progenitor cells. ^2^ human brown adipose progenitor cells. ^3^ human adipose progenitor cells. ^4^ human umbilical vein endothelial cells. ^5^ 4arm poly(ethylene glycol) acrylate.

**Table 8:** Muscle cell application, for 3D-Bioplotter™ technology.

3D Printer Used	Cell Line (s) Used	Materials Used	Applications	Reference
3D-Bioplotter™	L8 myoblasts and Schwann cells	Alginate hydrogel and DMEM ^1^	Characterization of cell damage and proliferative ability during and after bioprinting	[78]

^1^ Dulbecco’s modified eagle medium.

**Table 9:** Principal Schwann cell applications.

3D Printer Used	Cell Line (s) Used	Materials Used	Applications	Reference
3D-Bioplotter™	Living Schwann cells	Alginate/RGD ^1^-alginate hydrogel, hyaluronic acid, fibrinogen, and calcium chloride	Potential nerve tissue engineering applications	[78]
3D-Bioplotter™	Rat primary Schwann cells	Alginate hydrogel, RGD/YIGSR ^2^ peptides, and calcium chloride/PEI ^3^	Peptide-modified alginate scaffolds	[79]
3D-Bioplotter™	RSC96 cell line ^4^	Alginate hydrogel, hyaluronic acid, and calcium chloride	Scaffolds with high integrity and cell viability	[81]
3D-Bioplotter™	RSC96 cell line and L8 myoblasts	Alginate hydrogel and DMEM ^5^	Characterization of cell damage and proliferative ability during and after bioprinting	[78]
3D-Bioplotter™	RSC96 cell line	Alginate hydrogel and calcium chloride/PEI	Repair of peripheral nerve injury	[80]
3D-Bioplotter™	Rat Schwann cells and ATDC5 ^6^	Alginate-hyaluronic acid hydrogel and calcium chloride/PVA ^7^ or PEI ^8^	Tissue reparation	[75]

^1^ arginine-glycine-aspartate peptide. ^2^ tyrosine-isoleucine-glycine-serine-arginine peptide. ^3^ polyethyleneimine. ^4^ ATTC immortalized rat Schwann cell line. ^5^ Dulbecco’s modified eagle medium. ^6^ mouse teratocarcinoma cell line. ^7^ poly(vinyl alcohol). ^8^ polyethyleneimine.

**Table 10:** Principal skin tissue applications.

3D Printer Used	Cell Line (s) Used	Materials Used	Applications	Reference
3D-Bioplotter™	HDF ^1^ and HUVEC ^2^	35 formulations of PEG ^3^-X polymers	Cell-compatible hydrogels	[49]
3D-Bioplotter™	L929 fibroblasts and Human mesenchymal stem cells	Gelatin methacrylate hydrogel/alginate hydrogel and calcium chloride	Mesoscopic fluorescence tomography for bone tissue engineering	[70]
3D-Bioplotter™	NIH/3T3 cell line ^4^	Methacrylated gelatin hydrogel and EGF ^5^	Regenerative medicine for tympanic membrane perforations	[69]
3D-Bioplotter™	Primary human dermal fibroblast cells	Lignin—HPU ^6^ hydrogel	A new concept for fibroblasts bioprinting	[82]
3D-Bioplotter™	HEM ^7^, HaCat ^8^, and HDF	Gelatin methacrylamide hydrogel, collagen, and photoinitiator (and tyrosinase)	Bioprinting of living skin constructs	[83]

^1^ human dermal fibroblasts. ^2^ human umbilical vein endothelial cells. ^3^ poly(ethylene glycol). ^4^ murine fibroblast cell line. ^5^ epidermal growth factor. ^6^ hydrophilic polyurethane. ^7^ human melanocytes. ^8^ human keratinocytes.

**Table 11:** Some examples of different pressures applied to different cell types constructs, using a 3D-Bioplotter™ printer. All the pressures are expressed in kilopascals (kPa), to improve the comparison between articles.

Cell Line (s)	Pressure (kPa)	References
BMSCs ^1^	30–300	[56]
Primary chondrocytes (cartilage tissue)	10	[59]
hCPCs ^2^	70–80	[62]
Human iPSCs ^3^	5	[66]
ASMCs ^4^	300–350	[67]
hNSCs ^5^	150–200	[60]
ATDC5 ^6^	30	[58]
SaOS-2 cell line ^7^	90	[72,73]
21PT cell line ^8^	300–350	[67]
HWA ^9^ (+HUVEC ^10^)	300–350	[50]
Living Schwann cells	30	[78]
HDF ^11^ (+HUVEC)	100–250	[49]
Primary human dermal fibroblasts	200	[82]

^1^ bone marrow stromal cells. ^2^ human cardiac progenitor cells. ^3^ induced-pluripotent stem cells. ^4^ adipose-derived mesenchymal stem/stromal cells. ^5^ human neural stem cells. ^6^ mouse teratocarcinoma cell line. ^7^ sarcoma osteogenic cell line. ^8^ HER2 -positive breast tumour cell line. ^9^ human adipose progenitor cell line. ^10^ human umbilical vein endothelial cells. ^11^ human dermal fibroblasts.

## References

[1] Gabriel S., Hull C.W. (1984). Apparatus for production of three-dimensional objects by stereolithography. U.S. Patent.

[2] Melchels F.P., Feijen J., Grijpma D.W. (2010). A review on stereolithography and its applications in biomedical engineering. Biomaterials.

[3] Lee M.P., Cooper G.J.T., Hinkley T., Gibson G.M., Padgett M.J., Cronin L. (2015). Development of a 3D printer using scanning projection stereolithography. Sci. Rep..

[4] Lin D., Jin S., Zhang F., Wang C., Wang Y., Zhou C., Cheng G.J. (2015). 3D stereolithography printing of graphene oxide reinforced complex architectures. Nanotechnology.

[5] Hutmacher D.W., Schantz T., Zein I., Ng K.W., Teoh S.H., Tan K.C. (2001). Mechanical properties and cell cultural response of polycaprolactone scaffolds designed and fabricated via fused deposition modeling. J. Biomed. Mater. Res..

[6] Hafezi F., Kucukgul C., Ozler S., Koc B. (2015). Bioprinting: Application of Additive Manufacturing in Medicine. Additive Manufacturing.

[7] Gu Q., Hao J., Lu Y.J., Wang L., Wallace G.G., Zhou Q. (2015). Three-dimensional bio-printing. Sci. China Life Sci..

[8] Mullen L., Stamp R.C., Brooks W.K., Jones E., Sutcliffe C.J. (2009). Selective laser melting: A regular unit cell approach for the manufacture of porous, titanium, bone in-growth constructs, suitable for orthopedic applications. J. Biomed. Mater. Res. Part B Appl. Biomater..

[9] Nakamura M., Iwanaga S., Henmi C., Arai K., Nishiyama Y. (2010). Biomatrices and biomaterials for future developments of bioprinting and biofabrication. Biofabrication.

[10] Mueller B., Kochan D. (1999). Laminated object manufacturing for rapid tooling and patternmaking in foundry industry. Comput. Ind..

[11] Tappa K., Jammalamadaka U. (2018). Novel biomaterials used in medical 3D printing techniques. J. Funct. Biomater..

[12] Cui H., Nowicki M., Fisher J.P., Zhang L.G. (2017). 3D Bioprinting for Organ Regeneration. Adv. Healthc. Mater..

[13] Huang J.J., Ren J.A., Wang G.F., Li Z.A., Wu X.W., Ren H.J., Liu S. (2017). 3D-printed “fistula stent” designed for management of enterocutaneous fistula: An advanced strategy. World J. Gastroenterol..

[14] Horvath L., Umehara Y., Jud C., Blank F., Petri-Fink A., Rothen-Rutishauser B. (2015). Engineering an in vitro air-blood barrier by 3D bioprinting. Sci. Rep..

[15] Murphy S.V., Atala A. (2014). 3D bioprinting of tissues and organs. Nat. Biotechnol..

[16] Shukla M.R., Singh A.S., Piunno K., Saxena P.K., Jones A.M.P. (2017). Application of 3D printing to prototype and develop novel plant tissue culture systems. Plant Methods.

[17] Shi W., He R., Liu Y. (2015). 3D printing scaffolds with hydrogel materials for biomedical applications. Eur. J. Biomed. Res..

[18] Almeida C.R., Serra T., Oliveira M.I., Planell J.A., Barbosa M.A., Navarro M. (2014). Impact of 3-D printed PLA- and chitosan-based scaffolds on human monocyte/macrophage responses: Unraveling the effect of 3-D structures on inflammation. Acta Biomater..

[19] Hollister S.J. (2006). Porous scaffold design for tissue engineering. Nat. Mater..

[20] Hospodiuk M., Dey M., Sosnoski D., Ozbolat I.T. (2017). The bioink: A comprehensive review on bioprintable materials. Biotechnol. Adv..

[21] Drury J.L., Mooney D.J. (2003). Hydrogels for tissue engineering: Scaffold design variables and applications. Biomaterials.

[22] Campbell J., McGuinness I., Wirz H., Sharon A., Sauer-Budge A.F. (2015). Multimaterial and Multiscale Three-Dimensional Bioprinter. J. Nanotechnol. Eng. Med..

[23] Colosi C., Shin S.R., Manoharan V., Massa S., Costantini M., Barbetta A., Dokmeci M.R., Dentini M., Khademhosseini A. (2016). Microfluidic Bioprinting of Heterogeneous 3D Tissue Constructs Using Low-Viscosity Bioink. Adv. Mater..

[24] Vukicevic M., Mosadegh B., Min J.K., Little S.H. (2017). Cardiac 3D Printing and its Future Directions. JACC Cardiovasc. Imaging.

[25] Zhang Y.S., Yue K., Aleman J., Mollazadeh-Moghaddam K., Bakht S.M., Yang J., Jia W., Dell’Erba V., Assawes P., Shin S.R. (2017). 3D Bioprinting for Tissue and Organ Fabrication. Ann. Biomed. Eng..

[26] Blaeser A., Duarte Campos D.F., Puster U., Richtering W., Stevens M.M., Fischer H. (2016). Controlling Shear Stress in 3D Bioprinting is a Key Factor to Balance Printing Resolution and Stem Cell Integrity. Adv. Healthc. Mater..

[27] Jakus A.E., Rutz A.L., Shah R.N. (2016). Advancing the field of 3D biomaterial printing. Biomed. Mater..

[28] Skardal A., Atala A. (2015). Biomaterials for Integration with 3-D Bioprinting. Ann. Biomed. Eng..

[29] Chung J.H.Y., Naficy S., Yue Z., Kapsa R., Quigley A., Moulton S.E., Wallace G.G. (2013). Bio-ink properties and printability for extrusion printing living cells. Biomater. Sci..

[30] De Santis R., Gloria A., Russo T., D’Amora U., D’Antò V., Bollino F., Catauro M., Mollica F., Rengo S., Ambrosio L. (2013). Advanced composites for hard-tissue engineering based on PCL/organic-inorganic hybrid fillers: From the design of 2D substrates to 3D rapid prototyped scaffolds. Polym. Compos..

[31] Gurkan U.A., Tasoglu S., Kavaz D., Demirel M.C., Demirci U. (2012). Emerging technologies for assembly of microscale hydrogels. Adv. Healthc. Mater..

[32] Puppi D., Mota C., Gazzarri M., Dinucci D., Gloria A., Myrzabekova M., Ambrosio L., Chiellini F. (2012). Additive manufacturing of wet-spun polymeric scaffolds for bone tissue engineering. Biomed. Microdevices.

[33] Guvendiren M., Burdick J.A. (2013). Engineering synthetic hydrogel microenvironments to instruct stem cells. Curr. Opin. Biotechnol..

[34] Malda J., Visser J., Melchels F.P., Jüngst T., Hennink W.E., Dhert W.J.A., Groll J., Hutmacher D.W. (2013). 25th anniversary article: Engineering hydrogels for biofabrication. Adv. Mater..

[35] Hunt N.C., Grover L.M. (2010). Cell encapsulation using biopolymer gels for regenerative medicine. Biotechnol. Lett..

[36] Walters B.D., Stegemann J.P. (2014). Strategies for directing the structure and function of three-dimensional collagen biomaterials across length scales. Acta Biomater..

[37] Tasoglu S., Diller E., Guven S., Sitti M., Demirci U. (2014). Untethered micro-robotic coding of three-dimensional material composition. Nat. Commun..

[38] Bonino C.A., Efimenko K., Jeong S.I., Krebs M.D., Alsberg E., Khan S.A. (2012). Three-dimensional electrospun alginate nanofiber mats via tailored charge repulsions. Small.

[39] Jeon O., Alsberg E. (2013). Photofunctionalization of Alginate Hydrogels to Promote Adhesion and Proliferation of Human Mesenchymal Stem Cells. Tissue Eng. Part A.

[40] Kleinman H.K., Martin G.R. (2005). Matrigel: Basement membrane matrix with biological activity. Semin. Cancer Biol..

[41] Enam S. (2015). Substrates for clinical applicability of stem cells. World J. Stem Cells.

[42] Poldervaart M.T., Gremmels H., Van Deventer K., Fledderus J.O., Öner F.C., Verhaar M.C., Dhert W.J.A., Alblas J. (2014). Prolonged presence of VEGF promotes vascularization in 3D bioprinted scaffolds with defined architecture. J. Control. Release.

[43] Bourgine P.E., Scotti C., Pigeot S., Tchang L.A., Todorov A., Martin I. (2014). Osteoinductivity of engineered cartilaginous templates devitalized by inducible apoptosis. Proc. Natl. Acad. Sci. USA.

[44] Tasoglu S., Kavaz D., Gurkan U.A., Guven S., Chen P., Zheng R., Demirci U. (2013). Paramagnetic levitational assembly of hydrogels. Adv. Mater..

[45] Yang X., Sarvestani S.K., Moeinzadeh S., He X., Jabbari E. (2013). Three-Dimensional-Engineered Matrix to Study Cancer Stem Cells and Tumorsphere Formation: Effect of Matrix Modulus. Tissue Eng. Part A.

[46] Leung G.K.K., Wang Y.C., Wu W. (2012). Peptide nanofiber scaffold for brain tissue reconstruction. Methods in Enzymology.

[47] Cui X., Boland T. (2009). Human microvasculature fabrication using thermal inkjet printing technology. Biomaterials.

[48] Miller J.S., Stevens K.R., Yang M.T., Baker B.M., Nguyen D.H.T., Cohen D.M., Toro E., Chen A.A., Galie P.A., Yu X. (2012). Rapid casting of patterned vascular networks for perfusable engineered three-dimensional tissues. Nat. Mater..

[49] Rutz A.L., Hyland K.E., Jakus A.E., Burghardt W.R., Shah R.N. (2015). A multimaterial bioink method for 3D printing tunable, cell-compatible hydrogels. Adv. Mater..

[50] Qi D., Wu S., Kuss M.A., Shi W., Chung S., Deegan P.T., Kamenskiy A., He Y., Duan B. (2018). Mechanically robust cryogels with injectability and bioprinting supportability for adipose tissue engineering. Acta Biomater..

[51] Cui X., Breitenkamp K., Finn M.G., Lotz M., D’Lima D.D. (2012). Direct Human Cartilage Repair Using Three-Dimensional Bioprinting Technology. Tissue Eng. Part A.

[52] Kundu J., Shim J.H., Jang J., Kim S.W., Cho D.W. (2015). An additive manufacturing-based PCL-alginate-chondrocyte bioprinted scaffold for cartilage tissue engineering. J. Tissue Eng. Regen. Med..

[53] Esposito A.R., Moda M., Cattani S.M., de Santana G.M., Barbieri J.A., Munhoz M.M., Cardoso T.P., Barbo M.L.P., Russo T., D’Amora U. (2013). PLDLA/PCL-T Scaffold for Meniscus Tissue Engineering. Biores. Open Access.

[54] Calandrelli L., Immirzi B., Malinconico M., Luessenheide S., Passaro I., Di Pasquale R., Oliva A. (2004). Natural and synthetic hydroxyapatite filled PCL: Mechanical properties and biocompatibility analysis. Proceedings of the Journal of Bioactive and Compatible Polymers.

[55] Catros S., Fricain J.C., Guillotin B., Pippenger B., Bareille R., Remy M., Lebraud E., Desbat B., Amédée J., Guillemot F. (2011). Laser-assisted bioprinting for creating on-demand patterns of human osteoprogenitor cells and nano-hydroxyapatite. Biofabrication.

[56] Fedorovich N.E., De Wijn J.R., Verbout A.J., Alblas J., Dhert W.J.A. (2008). Three-dimensional fiber deposition of cell-laden, viable, patterned constructs for bone tissue printing. Tissue Eng. Part A.

[57] Kuss M.A., Harms R., Wu S., Wang Y., Untrauer J.B., Carlson M.A., Duan B. (2017). Short-term hypoxic preconditioning promotes prevascularization in 3D bioprinted bone constructs with stromal vascular fraction derived cells. RSC Adv..

[58] You F., Wu X., Zhu N., Lei M., Eames B.F., Chen X. (2016). 3D Printing of Porous Cell-Laden Hydrogel Constructs for Potential Applications in Cartilage Tissue Engineering. ACS Biomater. Sci. Eng..

[59] You F., Chen X., Cooper D.M.L., Chang T., Eames B.F. (2019). Homogeneous hydroxyapatite/alginate composite hydrogel promotes calcified cartilage matrix deposition with potential for three-dimensional bioprinting. Biofabrication.

[60] Gu Q., Tomaskovic-Crook E., Wallace G.G., Crook J.M. (2018). Engineering human neural tissue by 3D bioprinting. Methods Mol. Biol..

[61] Jakab K., Norotte C., Damon B., Marga F., Neagu A., Besch-Williford C.L., Kachurin A., Church K.H., Park H., Mironov V. (2008). Tissue Engineering by Self-Assembly of Cells Printed into Topologically Defined Structures. Tissue Eng. Part A.

[62] Bejleri D., Streeter B.W., Nachlas A.L.Y., Brown M.E., Gaetani R., Christman K.L., Davis M.E. (2018). A Bioprinted Cardiac Patch Composed of Cardiac-Specific Extracellular Matrix and Progenitor Cells for Heart Repair. Adv. Healthc. Mater..

[63] Izadifar M., Chapman D., Babyn P., Chen X., Kelly M.E. (2018). UV-Assisted 3D Bioprinting of Nanoreinforced Hybrid Cardiac Patch for Myocardial Tissue Engineering. Tissue Eng. Part C Methods.

[64] Visk D. (2015). Will Advances in Preclinical In Vitro Models Lower the Costs of Drug Development?. Appl. Vitr. Toxicol..

[65] Lewis P.L., Yan M., Su J., Shah R.N. (2019). Directing the growth and alignment of biliary epithelium within extracellular matrix hydrogels. Acta Biomater..

[66] Gu Q., Tomaskovic-Crook E., Wallace G.G., Crook J.M. (2017). 3D Bioprinting Human Induced Pluripotent Stem Cell Constructs for In Situ Cell Proliferation and Successive Multilineage Differentiation. Adv. Healthc. Mater..

[67] Wang Y., Shi W., Kuss M., Mirza S., Qi D., Krasnoslobodtsev A., Zeng J., Band H., Band V., Duan B. (2018). 3D Bioprinting of Breast Cancer Models for Drug Resistance Study. ACS Biomater. Sci. Eng..

[68] Narayanan L.K., Huebner P., Fisher M.B., Spang J.T., Starly B., Shirwaiker R.A. (2016). 3D-Bioprinting of Polylactic Acid (PLA) Nanofiber-Alginate Hydrogel Bioink Containing Human Adipose-Derived Stem Cells. ACS Biomater. Sci. Eng..

[69] Kuo C.-Y., Wilson E., Fuson A., Gandhi N., Monfaredi R., Jenkins A., Romero M., Santoro M., Fisher J.P., Cleary K. (2018). Repair of Tympanic Membrane Perforations with Customized Bioprinted Ear Grafts Using Chinchilla Models. Tissue Eng. Part A.

[70] Tang Q., Piard C., Lin J., Nan K., Guo T., Caccamese J., Fisher J., Chen Y. (2018). Imaging stem cell distribution, growth, migration, and differentiation in 3-D scaffolds for bone tissue engineering using mesoscopic fluorescence tomography. Biotechnol. Bioeng..

[71] Yu H.-Y., Ma D.-D., Wu B.-L. (2017). Gelatin/alginate hydrogel scaffolds prepared by 3D bioprinting promotes cell adhesion and proliferation of human dental pulp cells in vitro. Nan Fang Yi Ke Da Xue Xue Bao.

[72] Wang X., Tolba E., Der H.C.S., Neufurth M., Feng Q., Diehl-Seifert B.R., Mü Ller W.E.G. (2014). Effect of bioglass on growth and biomineralization of saos-2 cells in hydrogel after 3d cell bioprinting. PLoS ONE.

[73] Neufurth M., Wang X., Schröder H.C., Feng Q., Diehl-Seifert B., Ziebart T., Steffen R., Wang S., Müller W.E.G. (2014). Engineering a morphogenetically active hydrogel for bioprinting of bioartificial tissue derived from human osteoblast-like SaOS-2 cells. Biomaterials.

[74] Billiet T., Gevaert E., De Schryver T., Cornelissen M., Dubruel P. (2014). The 3D printing of gelatin methacrylamide cell-laden tissue-engineered constructs with high cell viability. Biomaterials.

[75] Rajaram A., Schreyer D.J., Chen D.X.B. (2015). Use of the polycation polyethyleneimine to improve the physical properties of alginate-hyaluronic acid hydrogel during fabrication of tissue repair scaffolds. J. Biomater. Sci. Polym. Ed..

[76] Olubamiji A.D., Izadifar Z., Zhu N., Chang T., Chen X., Eames B.F. (2016). Using synchrotron radiation inline phase-contrast imaging computed tomography to visualize three-dimensional printed hybrid constructs for cartilage tissue engineering. J. Synchrotron Radiat..

[77] DaSilva-Arnold S., Kuo C., Davra V., Remache Y., Cw P., Fisher J.P., Zamudio S., Al-khan A., Birge R.B., Nicholas P. (2019). ZEB2, a master regulator of the epithelial-mesenchymal transition, mediates trophoblast differentiation. Mol. Hum. Reprod..

[78] Ning L., Sun H., Lelong T., Guilloteau R., Zhu N., Schreyere D.J., Chen X. (2019). 3D bioprinting of scaffolds with living Schwann cells for potential nerve tissue engineering applications. Biofabrication.

[79] Sarker M.D., Naghieh S., McInnes A.D., Ning L., Schreyer D.J., Chen X. (2019). Bio-fabrication of peptide-modified alginate scaffolds: Printability, mechanical stability and neurite outgrowth assessments. Bioprinting.

[80] Rajaram A., Schreyer D.J., Chen X. (2012). Development of Schwann Cell-Encapsulated Alginate Scaffolds for the Repair of Peripheral Nerve Injury. CMBES.

[81] Rajaram A., Schreyer D., Chen D. (2016). Bioplotting Alginate/Hyaluronic Acid Hydrogel Scaffolds with Structural Integrity and Preserved Schwann Cell Viability. 3D Print. Addit. Manuf..

[82] Oveissi F., Naficy S., Le T.Y.L., Fletcher D.F., Dehghani F. (2018). Tough and Processable Hydrogels Based on Lignin and Hydrophilic Polyurethane. ACS Appl. Bio Mater..

[83] Shi Y., Xing T.L., Zhang H.B., Yin R.X., Yang S.M., Wei J., Zhang W.J. (2018). Tyrosinase-doped bioink for 3D bioprinting of living skin constructs. Biomed. Mater..

[84] Axpe E., Oyen M.L. (2016). Applications of alginate-based bioinks in 3D bioprinting. Int. J. Mol. Sci..

[85] Jia J., Richards D.J., Pollard S., Tan Y., Rodriguez J., Visconti R.P., Trusk T.C., Yost M.J., Yao H., Markwald R.R. (2014). Engineering alginate as bioink for bioprinting. Acta Biomater..

[86] Cuadros T.R., Erices A.A., Aguilera J.M. (2015). Porous matrix of calcium alginate/gelatin with enhanced properties as scaffold for cell culture. J. Mech. Behav. Biomed. Mater..

[87] Kulseng B., Skjåk-Braek G., Ryan L., Andersson A., King A., Faxvaag A., Espevik T. (1999). Transplantation of alginate microcapsules: Generation of antibodies against alginates and encapsulated porcine islet-like cell clusters. Transplantation.

[88] Pawar S.N., Edgar K.J. (2012). Alginate derivatization: A review of chemistry, properties and applications. Biomaterials.

[89] Schuurman W., Levett P.A., Pot M.W., van Weeren P.R., Dhert W.J.A., Hutmacher D.W., Melchels F.P.W., Klein T.J., Malda J. (2013). Gelatin-Methacrylamide Hydrogels as Potential Biomaterials for Fabrication of Tissue-Engineered Cartilage Constructs. Macromol. Biosci..

[90] Derby B. (2008). Bioprinting: Inkjet printing proteins and hybrid cell-containing materials and structures. J. Mater. Chem..

[91] Owens C.M., Marga F., Forgacs G., Heesch C.M. (2013). Biofabrication and testing of a fully cellular nerve graft. Biofabrication.

[92] Larocca R.A., Moraes-Vieira P.M., Bassi Ê.J., Semedo P., de Almeida D.C., da Silva M.B., Thornley T., Pacheco-Silva A., Câmara N.O.S. (2013). Adipose Tissue-Derived Mesenchymal Stem Cells Increase Skin Allograft Survival and Inhibit Th-17 Immune Response. PLoS ONE.

[93] Izadifar Z., Chang T., Kulyk W., Chen X., Eames B.F. (2015). Analyzing Biological Performance of 3D-Printed, Cell-Impregnated Hybrid Constructs for Cartilage Tissue Engineering. Tissue Eng. Part C Methods.

[94] Ning L., Betancourt N., Schreyer D.J., Chen X. (2018). Characterization of Cell Damage and Proliferative Ability during and after Bioprinting. ACS Biomater. Sci. Eng..

[95] Nair K., Gandhi M., Khalil S., Yan K.C., Marcolongo M., Barbee K., Sun W. (2009). Characterization of cell viability during bioprinting processes. Biotechnol. J..

[96] Kwok T.-H., Wang C.C.L., Deng D., Zhang Y., Chen Y. (2015). Four-Dimensional Printing for Freeform Surfaces: Design Optimization of Origami and Kirigami Structures. J. Mech. Des..

[97] Castro N.J., Meinert C., Levett P., Hutmacher D.W. (2017). Current developments in multifunctional smart materials for 3D/4D bioprinting. Curr. Opin. Biomed. Eng..

[98] Yang G.H., Yeo M., Koo Y.W., Kim G.H. (2019). 4D Bioprinting: Technological Advances in Biofabrication. Macromol. Biosci..

